# Home-based neurologic music therapy for upper limb rehabilitation with stroke patients at community rehabilitation stage—a feasibility study protocol

**DOI:** 10.3389/fnhum.2015.00480

**Published:** 2015-09-23

**Authors:** Alexander J. Street, Wendy L. Magee, Helen Odell-Miller, Andrew Bateman, Jorg C. Fachner

**Affiliations:** ^1^Music and Performing Arts, Music for Health Research Centre, Anglia Ruskin UniversityCambridge, UK; ^2^Music Therapy Program, Boyer College of Music and Dance, Temple UniversityPhiladelphia, PA, USA; ^3^Department of Psychiatry, University of CambridgeCambridge, UK; ^4^National Institute for Health Research, Collaborations for Leadership in Applied Health Research and Care, Cambridgeshire and Peterborough NHS TrustCambridge, UK; ^5^Oliver Zangwill Centre for Neuropsychological RehabilitationEly, UK; ^6^Cambridgeshire Community Services NHS TrustSt Ives, UK

**Keywords:** stroke, hemiparetic, therapeutic instrumental music performance (TIMP), music-supported therapy, ARAT, community rehabilitation, feasibility study

## Abstract

**Background:** Impairment of upper limb function following stroke is more common than lower limb impairment and is also more resistant to treatment. Several lab-based studies with stroke patients have produced statistically significant gains in upper limb function when using musical instrument playing and techniques where rhythm acts as an external time-keeper for the priming and timing of upper limb movements.

**Methods:** For this feasibility study a small sample size of 14 participants (3–60 months post stroke) has been determined through clinical discussion between the researcher and study host in order to test for management, feasibility and effects, before planning a larger trial determined through power analysis. A cross-over design with five repeated measures will be used, whereby participants will be randomized into either a treatment (*n* = 7) or wait list control (*n* = 7) group. Intervention will take place twice weekly over 6 weeks. The ARAT and 9HPT will be used to measure for quantitative gains in arm function and finger dexterity, pre/post treatment interviews will serve to investigate treatment compliance and tolerance. A lab based EEG case comparison study will be undertaken to explore audio-motor coupling, brain connectivity and neural reorganization with this intervention, as evidenced in similar studies.

**Discussion:** Before evaluating the effectiveness of a home-based intervention in a larger scale study, it is important to assess whether implementation of the trial methodology is feasible. This study investigates the feasibility, efficacy and patient experience of a music therapy treatment protocol comprising a chart of 12 different instrumental exercises and variations, which aims at promoting measurable changes in upper limb function in hemiparetic stroke patients. The study proposes to examine several new aspects including home-based treatment and dosage, and will provide data on recruitment, adherence and variability of outcomes.

## Background

There are approximately 152,000 people affected by stroke in the UK every year (British Heart Foundation, 2012) causing more disability in adults than any other disease or condition. More than 50% of these report severe disability (Adamson et al., 2004b) and face long-term dependency on others for support with daily activities in their home (Adamson et al., 2004a). The mean length of stay in hospital for stroke patients in the UK has fallen from 32 days in 2000 to 20 days in 2010 (British Heart Foundation, 2012). Community services, sometimes referred to as “early supported discharge teams,” and other community based rehabilitation teams are reported to improve outcomes for stroke patients, but an audit in 2010 recorded only 36% of hospitals in the UK were providing such services (Department of Health, 2010). A shortfall in spending on chronic stroke rehabilitation is also reported in the US (Miller et al., 2010), despite the fact that studies have shown improvements in outcomes for patients when interventions continue from acute care into the community up to five years after stroke (Fens et al., 2013).

Weakness on one side, or hemiparesis, is the most commonly encountered sensorimotor impairment following ischaemic or haemorrhagic stroke (Sabini et al., 2013), occurring in 80% of patients (Adey-Wakeling and Crotty, 2013). Hemiparesis has a profound effect on patients' ability to perform ADLs such as washing, dressing, cooking and eating, and is extremely resistant to rehabilitation treatments. The total financial costs resulting from stroke in 2009, including direct health care costs, productivity loss and informal care were £3,741,682 (British Heart Foundation, 2012). Other estimates put the annual cost figure at 7 billion with 2.8 billion comprising direct healthcare costs (Bhatnagar et al., 2010).

Research beginning in the 1990s into rhythm driven interventions for gait training following stroke and traumatic brain injury (Thaut et al., 1993, 1997, 2007; Prassas et al., 1997; Hurt et al., 1998), in Parkinson's disease (Thaut et al., 1996; McIntosh et al., 1997), and with cerebral palsy (Kwak, 2007; Kim et al., 2011, 2012) has resulted in a well evidenced intervention known as Rhythmic Auditory Stimulation (RAS). RAS is reported to improve gait parameters including stride length and symmetry with stroke patients, with further research recommended into rhythm driven interventions in neurorehabilitation (Bradt et al., 2010). Building on this research Thaut et al. (2002) and Malcolm et al. (2009a) found evidence for the application of rhythm driven interventions in upper limb rehabilitation, with participants making significant improvements in movement trajectories and quality of arm movement. Motivation is a major factor that, when lacking, can hinder engagement in rehabilitation programs, and a number of other studies illustrate the use of music and the inclusion of music therapy within multidisciplinary rehabilitation in order to improve patient mood and enhance motivation (Nayak et al., 2000; Jochims, 2004; Magee et al., 2006; Craig, 2008; Sarkamo et al., 2008; Magee and Baker, 2009; Street, 2012). Using electronic drums supported with live music from the music therapist, Paul and Ramsey (1998) found clinical (but not statistical) significance in increased active shoulder and elbow range for stroke participants. Sharing some features with this study, Music Supported Therapy (MST) is a recently researched intervention in which participants played through a series of increasingly complex musical exercises using electronic drum pads and keyboard. Results from these studies have consistently shown statistically significant improvements for participants' upper limb function, also evidencing neural reorganization using EEG and fMRI technology (Schneider et al., 2007; Rojo et al., 2011; Altenmüller et al., 2009; Grau-Sánchez et al., 2013). EEG was recorded during playing, i.e., hitting a key or a drum pad, which would indicate an event in the EEG. Pre-post therapy results in the music group showed an increase of Event-Related-Desynchronization and coherence in the beta band indicating reorganization of motor patterns (Altenmüller et al., 2009). Rojo et al.'s case study indicated that music patterns that were listened to before they were played by participants showed increased activation of motor and auditory regions when listening after the patterns had been learned, at the end of treatment (Rojo et al., 2011). Evidence suggesting that the music generated by the participants' playing during these studies has induced neural reorganization, whereby the auditory cortices appear to be incorporated into motor circuits, has prompted use of the term “audio-motor coupling” (Rojo et al., 2011), a phenomenon also observed within minutes of novice piano players beginning to practice (Classen et al., 1998). Musical motor performance involves the same brain regions as other motor tasks, those being the: motor, premotor, supplementary motor area (SMA), the cerebellum and the basal ganglia, as well as somatosensory, auditory, emotional, temporal, and memory loops (Altenmüller, 2001; Lotze et al., 2003; Meister et al., 2004). Musicians perform complex movement patterns, which are informed by continuous auditory feedback from their playing (Altenmüller et al., 2009), and feedback from movements is fundamentally important in order to inform and control them (Carpenter and Reddi, 2012).

Therapeutic Instrumental Music Performance (TIMP) is a Neurologic Music Therapy intervention (NMT) used in neurorehabilitation which employs external audio cues during music based activities in which the selection and spatial arrangement of instruments facilitates improved upper limb movement trajectories and arm kinematics (Thaut, 2008). Jeong and Kim (2007) suggest that the combination of rhythmic music and movement attuned to it creates a powerful neurological stimulus that may increase the plasticity of the nervous system. TIMP is one of a number of NMT interventions applied to sensorimotor, cognitive and communication rehabilitation (Thaut, 2008). It involves the planning of functional therapeutic musical experiences to meet functional physical goals, set within the multidisciplinary team, with the aim of transferring the therapeutic learning into real-world applications. Whilst evidence has emerged from the aforementioned studies regarding the effects of either rhythm or musical instrument playing on neural reorganization and upper limb movement trajectories, there have been very few that combine these elements to form a unified treatment protocol matching that of TIMP. Lim et al. (2011) investigated its effects on perceived exertion and fatigue, with positive findings, but did not measure for any physiological change. Paul and Ramsey's (1998) study matches the TIMP protocol, but was delivered in a group setting. Yoo (2009) conducted a study using TIMP in a lab setting with three chronic stroke patients and found evidence of improved wrist and hand function, as well as increased movement velocity.

Music therapy is not commonly associated with, nor found within, neurorehabilitation settings; in 2005 only four neurorehabilitation units in the UK employed a music therapist (Magee et al., 2006). Musical instrument playing is not widely recognized as a feasible and effective, short-term intervention for treating movement disorders resulting from stroke, a patient group within which there is a high level of heterogeneity as regards upper limb hemiparesis, cognitive, sensory and communication impairments. Yoo's study, which included three participants, was conducted at Colorado State University, a major center for NMT research and training. Participants were recruited from a facility managed by their center for biomedical research in music. Heterogeneity influences decisions regarding inclusion criteria; if it is too specific, then recruitment can be slow, too broad and heterogeneity introduces more variables, which in turn may skew statistical outcomes. In either case, a further influence is the pool size from which patients will be recruited; the geographical area and whether single or multi-site.

Home based and combined home/clinic training programs for sensorimotor treatment have been trialed previously using RAS gait training with Parkinson's patients (Thaut et al., 1996), rhythmic auditory cueing for upper limb reaching kinematics with stroke patients (Malcolm et al., 2009b), and computer gaming (King et al., 2012), however, all other research relating to this topic has been laboratory based. There is a lack of research investigating sensorimotor interventions with patients at the home-based community stage of rehabilitation. Previous studies investigating musical instrument playing have included in-patients, who were, on average, approximately 2 months post-stroke (Schneider et al., 2007; Altenmüller et al., 2009; Grau-Sánchez et al., 2013). One study using a rhythm and music-based therapy program included participants at 1–5 years post stroke (Bunketorp Kall et al., 2012). This study will include participants 3–60 months post stroke, defined as being at the chronic stage of recovery (Barrett and Meschia, 2013).

Frequency of therapy sessions in existing studies has been predominantly 5 days per week for 3–4 weeks (Schneider et al., 2007; Altenmüller et al., 2009; Malcolm et al., 2009a; Rojo et al., 2011; Amengual et al., 2013), which is comparable with typical modified constraint induced movement therapy (mCIMT) delivery (Earley et al., 2010), and the music therapy treatment has usually been compared with other forms of standard care or combined music therapy/standard care. Early versus late treatment using RAS in gait training has been trialed (Hayden et al., 2009), but music therapy treatment for upper limb rehabilitation has not been investigated using a wait list design. The feasibility of delivering RAS for stroke patients as part of standard care has been explored (Hayden et al., 2009). Owing to the innovative nature of this intervention and recruitment of participants from within an NHS trust where neurologic music therapy is not recognized or available, participants will be recruited after discharge from community stroke rehabilitation services.

The study reported here will build upon the existing knowledge of music's effect on neuroplasticity (Schneider et al., 2007; Altenmüller et al., 2009; Rojo et al., 2011; Amengual et al., 2013) and translate this knowledge into a clinical protocol that may improve patient outcomes. Thus, it will add to limited, existing research into musical instrument playing, rhythm and upper limb rehabilitation following stroke. It will also address questions concerning dosage, setting and the timing of treatment delivery. Whereas most of the research to date on this topic has been laboratory based, this study provides a novel intervention that will be delivered one-to-one, in participants' homes. It will therefore examine the feasibility of home treatment delivery at the end of standard community care. In addition, participant experience of TIMP recorded via semi-structured interview will provide data regarding motivation, access and compliance to treatment. Frequency of sessions will be reduced compared to previous studies, in order to determine whether it is still effective at a lower dosage and to ensure that the sample size can be treated within the timeframes and resources available for this research.

### Study aims and objectives

The aims of this crossover study are to investigate whether TIMP is a feasible and effective home-based intervention for upper limb hemiparesis following stroke, when delivered at a frequency of twice weekly for a period of 6 weeks. Additional qualitative data gathered will explore the participant experience of this treatment with specific focus on feasibility of treatment delivery in the home, patient motivation, and patient preference with regard to the treatment methods under investigation. This biomedical research study is registered with clinical trials.gov, number NCT 02310438 and also approved by the Integrated Research Application System (IRAS) and Anglia Ruskin University Ethics Boards.

## Methods

### Study design

**Figure 1:**
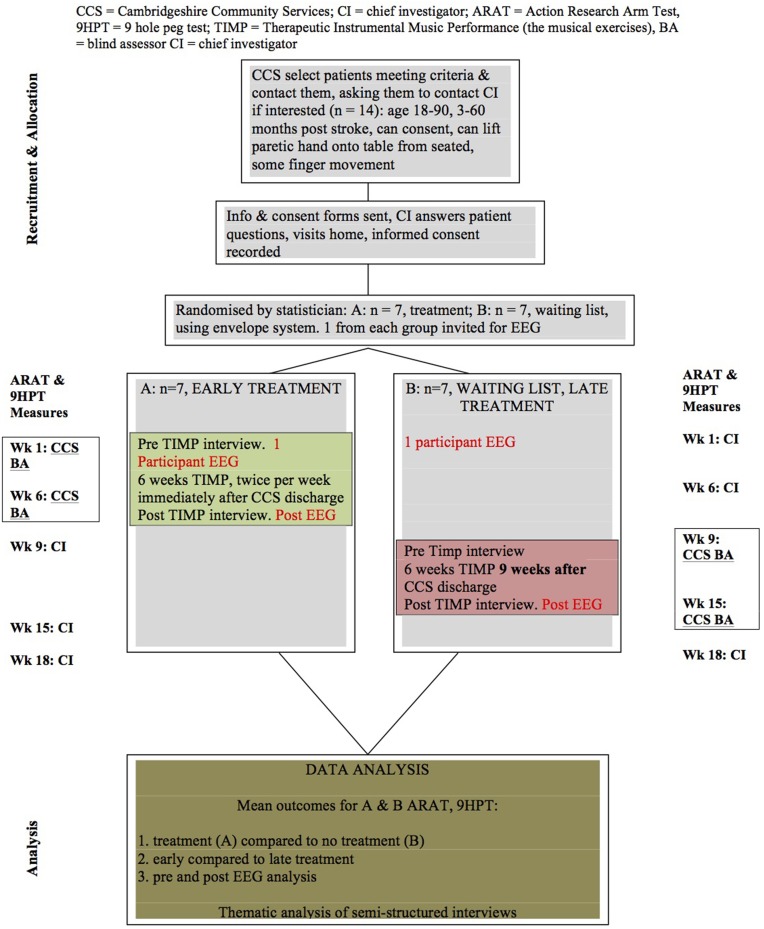
**Study consort, showing the crossover design with repeated measures**. BA, Blind Assessor, who will conduct ARAT and 9HPT indicated at Weeks 1 and 6 (treatment), 9 and 15 (wait list).

A cross-over design with repeated measures will be used, with participants being randomized into either a wait list or treatment group (see Figure 1). Assessments for each participant will take place at the same time points after baseline measure, as illustrated in Figure 1, with the baseline and end assessments immediately before and after the 6 weeks of TIMP being conducted by a therapist who is blind to participant allocation.

### Procedure

Each participant will have a total of 12 individual music therapy sessions in their home, delivered twice weekly over the course of 6 weeks. They will not be required to perform any practice or exercises set by the researcher between sessions. Whilst in wait list, before the intervention begins, participants will not receive any community nor privately employed rehabilitation interventions for upper limb hemiparesis. Each participant will also receive the Action Research Arm Test (ARAT) (Lyle, 1981) and Nine Hole Peg Test (9HPT) (Kellor et al., 1971) assessments at the same five time points after baseline measure, over an 18 week period as follows: Timepoint 1 at Week 1 after randomization; timepoint 2 at week 6; timepoint 3 at week 9; timepoint 4 at week 15; and timepoint 5 at week 18. The design will allow analysis of treatment and no treatment by comparing treatment group with wait list group data. It will also be possible to compare early versus late intervention following discharge from the community rehabilitation treatment, as the wait list participants will have a delay of 9 weeks between community discharge and beginning TIMP. Data collected from wait list group participants prior to TIMP treatment will be analyzed to determine whether there has been any spontaneous change in upper limb function, which can occur as an independent covariate (Kwakkel et al., 2006). Subject data from the ARAT and 9HPT will also be individually analyzed in order to discuss group results.

Although unusual in a randomized cross-over design study, qualitative data will be collected from each participant in order to explore patient preference with regard to using music to support exercises, and factors that might provide insights into patient tolerance and compliance. These aspects are important given the innovative treatment being used, the dosage and the setting i.e., within the home environment. Qualitative data will be collected by the researcher immediately before and after the 6 weeks of TIMP, by using a semi-structured interview that has been devised for this study and comprises five questions regarding the participant's experience of playing the instruments and playing to the music. This data will provide an overall impression of the feasibility for this treatment protocol. Participant responses to the post semi-structured interview will specifically provide data regarding motivational effects. In addition, the researcher will gather information in a research journal during and after each session, which will describe emotional responses; no direct quotes from participants will be used. There is also a five point Likert scale for recording how much participants feel the treatment will help them and, at the end of the 6 weeks of TIMP, how much they feel it actually has helped them in their ADLs. The researcher will record, in written form, participants' responses during the interview for later thematic analysis. Open questions will be used to give participants the opportunity to express their preconceptions and communicate their experience of the treatment, with the possibility of other themes arising. The quantitative data gained with the Likert response scale is used to record a time series of treatment responses (control for auto-correlation is not intended).

### Recruitment

Participants will be recruited from three geographically separate community stroke rehabilitation teams in the south of England. Patients discharged from community rehabilitation who meet the inclusion criteria will be invited by the host NHS trust to participate in this study. It will not be possible to control for the length of time that each participant receives statutory community rehabilitation prior to joining the study, as within the host NHS trust this is extremely variable. Music therapy is also not recognized as an intervention for upper limb hemiparesis following stroke in the UK. As such, the host NHS trust cannot agree to facilitate any disruption or adjustment to standard treatment for their patients for music therapy research purposes. Such bodies as the Care Quality Commission (CQC) (Care Quality Commission, 2011) and National Institute for Clinical Excellence (NICE) (National Institute For Health and Care Excellence, 2013) do not recommend music therapy as an intervention with stroke patients. However, following a review of music therapy and traumatic brain injury by the Cochrane library, music therapy is now listed as a possible intervention within neurorehabilitation (Bradt et al., 2010). Potential participants who meet the inclusion criteria and, following invitation by the host NHS trust, have expressed an interest in the study will be visited in their home by the researcher in order to demonstrate the treatment methods, including the playing patterns, and answer any questions. All participants will be required to give informed consent, which will be recorded.

### Participants

Fourteen adult participants, 3–60 months post stroke, with hemiparesis will be recruited who have been discharged from community rehabilitation and can consent to treatment. The age range for inclusion will be 18–90. As with the MST studies (Schneider et al., 2007; Altenmüller et al., 2009; Rojo et al., 2011; Amengual et al., 2013) participants must be able to lift their affected hand up on to a table whilst seated, unaided by their unaffected side and have some finger movement in the affected hand. In a meta-analysis of literature reporting prognostic variables in upper limb recovery (Coupar et al., 2012), inconclusive evidence was found for time since stroke being a predictor. Comparisons between lesion site also revealed no predictive value in upper limb recovery. The most significant findings were that patients recovered significantly more upper limb function if there was less impairment in their upper limb initially caused by the stroke. The inclusion criteria for this feasibility study has been determined based on this research and that set for the MST studies earlier cited. On entry into the study participants will be randomized to either the treatment or wait list group, with a list of numbers randomly generated by an independent statistician using a computer, which will be concealed using opaque sealed envelopes. The assessor will be blinded to participant allocation. In order to maintain blinding the researcher will deliver a script for all participants and the assessor at each assessment time-point.

### Sample size

The sample size of 14 stroke patients was calculated not based on a power calculation but through discussion of clinical matters between the researcher and the NHS trust hosting the study. The discussion was based on defining an appropriate number of stroke patients that would represent the heterogeneity of upper limb impairment and facilitate a report on the feasibility, management and efficacy of the intervention under the stated research conditions (unknown music based treatment, in participants' homes, twice weekly). The sample size is considered feasible for the researcher to deliver with the available time scale and resources within the host NHS trust, including staff assisted access to patient records, identification of suitable patients and invitation to participate from NHS community stroke team therapists, and completion of data collection within a limited period of time. Risks, benefits and the logistics of delivering treatment in the home, given that it is labor intensive and delivered by the researcher alone, will be observed and reported, together with participant compliance. The home environment will introduce variables that cannot be controlled for, such as space available to set up equipment, management of seating equipment to optimize positioning for the intervention, distractions in sessions such as the activities of family members or other residents present in the home. The instruments will not be set up permanently, as would be the case in a research lab, where instrument height and distance from participant can be maintained and standardized. In this study TIMP will be delivered at a frequency much lower than has been the case in existing research of this nature, so effects at this dosage are not known. All of these logistical factors warrant detailed examination in a smaller feasibility study prior to moving to a study with a larger number of participants.

### Measures

**Figure 2:**
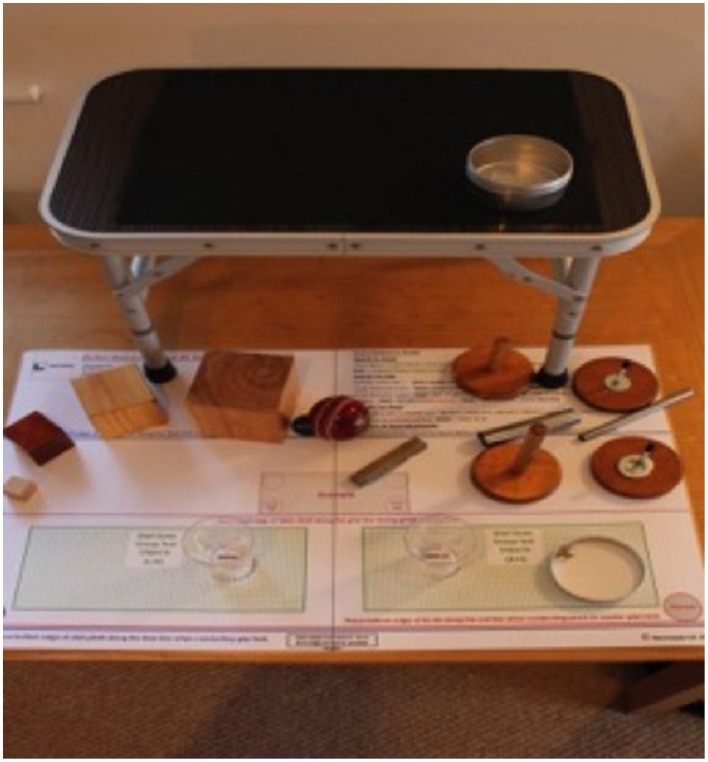
**The action research arm test**.

A wide range of assessment tools and technology has been used to record outcomes in upper limb post-stroke rehabilitation. The selection of assessment tools for this feasibility study has been determined by two major factors: (1) the restrictions enforced by the ethics committee who approved the study according to UK ethics procedures for research within the NHS via the Integrated Research Application System (IRAS), (2) availability of assessment tools and training requirements for their application. The Action Research Arm Test (ARAT), will be the primary outcome measure for this study; 9HPT will measure finger dexterity and semi-structured interviews allow for the collection of qualitative data on the participant experience. The ARAT has also been used in constraint-induced movement therapy studies (Kitago et al., 2013), and a study using rhythm and music with stroke patients (Bunketorp Kall et al., 2012). It has excellent inter-rater reliability (Hsieh et al., 1998; van der Lee et al., 2002), excellent intra-rater reliability (van der Lee et al., 2002), and excellent convergent validity against the Fugl-Meyer (De Weerdt, 1985). The ARAT is a timed, 19 item measure that is divided into four categories: grasp, grip, pinch and gross movement. Each item, or action, is performed by the participant while seated with their back against the chair, a measured distance from the table (15 cm), where all of the assessment items are individually placed for each task (see Figure 2). The test recreates the movements or sequences of movements required to perform many ADLs, such as reaching up onto a shelf to obtain a food ingredient or pouring liquid from one container to another. A table map for the ARAT is laid out flat on the table top and this has markers on it to indicate the start and end position for each object used in the test, thus optimizing consistency between patients and settings. The assessment can take up to 30 min if the patient needs to complete all items in every subcategory.

The 9HPT, more specifically measuring finger dexterity, is widely used in stroke rehabilitation and related research (Kellor et al., 1971). It has excellent inter-rater reliability and adequate intra-rater reliability (Oxford Grice et al., 2003). It is a timed test, using standardized equipment comprising a rectangular plastic tray with a rounded, concave tray at one end containing nine small white pegs and at the other end nine holes into which the participant must (one at a time) place the nine pegs from the tray and then remove them, placing them back into the tray as quickly as possible. The participant practices with the unaffected hand first, then the affected side. The participant is seated while performing the test, which takes no more than 2 minutes to administer, depending on the age of the patient and their existing degree of finger dexterity.

### Electroencephalography (EEG) recording

The EEG recording procedure will include rest, listening to the TIMP music patterns (that will be learned in the sessions) a Go/No go task, after which another resting state EEG will be recorded. This study will utilize a BrainAmp DC (Brainproducts) EEG utilizing 64 active electrodes and artifact channels (EOG, EMG). Artifact control will be guided by video recording of the participant whilst undergoing EEG. The video footage will inform about body movement and eye-blinks. After visual inspection and indexing of the continuous EEG traces, data pre-processing will utilize the Neuroguide Artifact toolbox (Thatcher et al., 2009) for the resting EEG and the music listening task. EEGLAB and ePrime will be used for the analysis of the Go/No Go task and will focus on the short episodes around the clicks. After visual inspection ICA will be applied to pre-process data and determined artifacts will be excluded.

### Semi-structured interviews

Using the principles and processes described in Interpretative Phenomenological Analysis (IPA) (Smith and Osborn, 2008) material from the semi-structured interviews will be analyzed into themes. The questions have been designed as open questions to encourage participants to offer descriptions of their experience of playing the instruments and playing to the supporting music. In keeping with IPA principles, the underlying aim of the questions is to offer participants a chance to explain how they feel the treatment will affect them and describe how they are experiencing, feeling and thinking about the processes involved, as such this will provide data regarding motivation and emotional response. Patient feedback in these areas will also feed into questions around feasibility of delivery for this treatment protocol.

### Statistical analysis

For the primary outcome measures, for the appropriate analysis of the crossover design with repeated measures, a linear mixed model approach will be used. This will be undertaken using the computer program R and will employ the R package lme4, which is sufficiently flexible to provide detailed analysis for this type of design, including the accommodation of missing values. The main result of this analysis will be the assessment of whether the music therapy has had an effect. To avoid the need to make strong assumptions about the distribution of the data, namely, that fitted model residuals are Normally distributed, computer-intensive methods will be employed for the statistical inferences. This will include bootstrap approaches to calculating 95% confidence limits, and permutation tests to obtain statistical significance test *P*-values.

## EEG analysis

EEG case analysis of the pre/post *resting state* EEG recordings will be treated with NeuroGuide Software (www.appliedneuroscience.com; Version 2.6.6) including an age, gender and condition-matched (*N* = 678 matched controls) LORETA normative EEG database (Thatcher et al., 2005). Continuous, artifact-free, raw EEG data will be subjected to a power spectral analysis (PSA) to calculate raw and z-scored spectral values, topography (Absolute power and Current Source Density), electrode correlation, burst metrics (burst number, amplitude, duration, and interval), instantaneous connectivity and coherence patterns, especially beta coherence. Due to the small sample size (*N* = 2) we do not expect significant differences between subjects' resting state displayed on central and temporal leads in beta power *z*-score topography and coherence data in the early compared to delayed intervention. However, we do expect intra-subject pre/post intervention z-score decreases in temporal and central leads. Topography will explore post-therapy spectral power in-, or decreases in central and temporal areas; coherence data of central and temporal leads will inform about post-therapy connectivity decreases or increases between motor and auditory cortex (as seen in beta coherence; Altenmüller et al., 2009). Pre/post intervention paired *t*-test will indicate the probabilities and directions of change. We expect the post-intervention measures to show a lowering of *z*-scores, i.e., normalization, and an increase of brain connectivity between central and temporal regions.

Low Resolution Tomography (LORETA), a specific mathematical solution to EEG source localization (Pascual-Marqui et al., 1994), will inform about raw and z-transformed current density means and their pre/post differences (subtraction/individual paired *t*-test) especially of the beta range. Further raw and z-transformed Region of Interest (ROI) correlations of primary and pre-motor [Brodmann Area (BA) 4,6, Pre central gyrus], auditory (BA 22, 41, 42, superior and transversal temporal gryus) and frontal (BA 44, 45, inferior frontal gyrus) cortices will inform about post-treatment related neural reorganization in audio-motor coupling, expected to be shown as increased ROI correlations.

A previous study observed post therapy changes in motor and auditory activity during the music listening (Altenmüller et al., 2009; Rojo et al., 2011). In this study we will compare the pre/post *music listening data* of TIMP patterns employed in the intervention. In Rojo's study, when the participant listened to the music played in sessions after the MST treatment period, they displayed an increase of motor and auditory responses when compared to listening before they had received any MST. We hope to demonstrate the same tendencies by utilizing topographic EEG mapping and LORETA. Differences in power means and probabilities of change will inform us about differences in responses to the particular TIMP patterns.

We will analyse the particular raw pre/post means and differences of listening sequences (see Table 1) on topography shifts (Absolute power and CSD), electrode correlation (PCC), burst metrics, instantaneous connectivity, phase and coherence patterns in particular on the beta range (Altenmüller et al., 2009). Altenmüller further investigated alpha ERD/S measured after hitting a trigger pad and was able to show differences in the response latencies. Increases in frontal midline theta (FMT) triggered by emotional responses during music listening have been reported in several music therapy studies (Sammler et al., 2007; Lee et al., 2012; Fachner et al., 2013; O'Kelly et al., 2013). We expect to see more increases in FMT post TIMP treatment (6 weeks). We will apply LORETA ROI correlation of beta, alpha and theta frequency power means for each TIMP pattern (see Table 1) to indicate task related pre-post changes in auditory, frontal and motor regions (see rest EEG analysis).

**Table 1:** **TIMP chart**.

	**Target movement (bold) and associated muscle groups**	**Instrument/s and equipment**	**Positioning**	**Participant instructions**	**Playing pattern**	**Facilitating music**	**Variations**
1	Single beat with affected side: **Elbow and shoulder flexion and extension, shoulder abduction and adduction**. Some wrist extension or deviation, finger extension, wrist between pronation and supination, core muscles	**14” Cymbal on boom stand**, adaptive beater and wooden drum stick, or finger picks slotted over thumb and/or finger/s	Cymbal positioned at a distance and height that the participant can reach to play Gradually raise height/increase distance and angle to facilitate increased movement range	Relax shoulders, feet flat as possible on floor/foot rests. Extend arm and fingers to cymbal. Relax and rest hand on lap or by side after playing Prompt can be sung to music: “reach and reach and play, relax”	Affected side always play on beat 3 of each bar	C, C, Am, Am, F, G, C, C 8 bar chord sequence in 4/4 Arpeggiating and building the intensity of each chord toward beat 3 Each cycle of the sequence requires 8 beats of the cymbal	**A**: using hand/fingers/finger picks **B**: adaptive beater or drum stick **C**: alternating affected and unaffected side (bell of cymbal on one beat)
2	2 successive beats with affected side: **Elbow flexion and extension**, slight shoulder extension and adduction, wrist between pronation and supination, grip, core muscles	**Cymbal on boom and bongos on stand,** adaptive beater and 2 X wooden drum sticks, finger picks	Bongos on affected side, at achievable height and angled so that larger one is further from participant, cymbal for unaffected side. Gradually reposition for increased range of movement	Try to focus on elbow bending and stretching Relax shoulders, feet flat as possible on floor, reach arm (and fingers). Relax arm and rest hand on lap after playing Sung prompt: Left (affected) and left and right, relax	In 6/8, play on the underlying pulse: affected hand plays bongo 1, then 2	Rhythmic, energetic, jazz idiom: G7, G#Dim, Am7, D7, played in 6/8 over 4 bars. Arpeggiated and/or strummed with strongly accented beats Each cycle of chords requires 6 beats on the percussion (4 on affected side)	**A:** using hand/fingers/finger picks **B:** adaptive beater (affected) drum stick (unaffected) **C:** drum stick both hands
3	3 successive beats with affected side: **Shoulder and elbow flexion, and extension**, **shoulder abduction**, slight shoulder adduction, wrist between pronation and supination, grip, core muscles	**Cymbal on boom and bongos on stand,** adaptive beater and wooden drum sticks, finger picks	Bongos on affected side (as above), adjusting the height and positioning of instruments to facilitate greater shoulder and elbow extension or shoulder abduction	Try to focus on elbow bending and stretching Sung prompt: Left (affected) and left and left and right	In 6/8, play on underlying pulse: affected side plays bongo 1, then 2, then cymbal, then unaffected side plays cymbal bell (hitting the centre of the cymbal)	Any 1, 6, 2, 5 chord sequence played over 4 bars. Strongly pulsed arpeggios or strummed rhythm in 6/8 Each cycle of chords requires 8 beats on the percussion (6 on affected side)	**A:** using hand/fingers/finger picks **B:** adaptive beater (affected side) and drum stick (unaffected) **C:** two drum sticks
4	4 successive beats with affected side: **Shoulder and elbow flexion and extension**, **shoulder abduction**, slight shoulder adduction, wrist between pronation and supination, grip, core muscles	**Cymbal on boom and bongos on stand,** adaptive beater and wooden drum sticks, finger picks	Bongos on affected side (as above), adjusting the height and positioning of instruments to facilitate greater shoulder and elbow extension or shoulder abduction	Try to focus on elbow bending and stretching Sung prompts as TIMP 2 and 3	In 6/8, on underlying pulse: affected side plays bongo 1, then 2, then cymbal, then cymbal bell, unaffected side then plays the pattern in reverse order	Any 1, 6, 2, 5 arpeggiated or strummed chord sequence played over two bars, strongly pulsed rhythm in 6/8 Each cycle of chords requires 8 beats from the affected side	**A:** using hand/fingers/finger picks **B:** two drum sticks, affected side plays the pattern, then unaffected side plays pattern in reverse while affected side rests
5	Bilateral playing, crossing midline: **Shoulder adduction, extension and flexion**, **elbow flexion and extension**, grip, wrist between pronation and supination, core muscles	**Cymbal on boom and bongos on stand,** adaptive beater and wooden drum sticks, finger picks	Bongos on affected side, cymbal on unaffected side Distance of instruments from participant and width apart of instruments varies, to facilitate increases in shoulder adduction and extension	Feet flat as possible on floor, try to relax shoulders and achieve smooth twisting action at shoulders and torso to play left and right	Both hands crossing midline alternating affected and unaffected side on beat 3 of each bar, progressing to beats 1 (L) and 3 (R)	Open “A” bass note played over arpeggiated or strummed chords: A, Bm, C#m, Bm, Played with clear pulse, 4/4 time over two bars	**A:** using hand/fingers/finger picks **B:** adaptive beater (affected) and drum stick (unaffected) **C:** two drum sticks
6	Fine motor: thumb only or thumb and index, middle or ring finger gripping a plectrum **Thumb extension and flexion**	**Tablet and Garageband** music software using “Smartguitar,” tablet touch screen plectrum, speaker connected	On lap on affected side, on stand at appropriate height, or on table top	Fingers rest on the side of the tablet, the thumb extends side to side across the screen strings Participants aim to strum across as many strings as possible with each thumb stroke, in time to the beat	Chord strummed using thumb only on beat 1 and 3 until final bar (C), which is beat 1 only	C, F, G, C sequence in 4/4. Each played with bass “lead in” as follows: g, a, b, **C** (*chord*), c, d, e, **F** (*chord*), f, e, d, **G** (*chord*), g, a, b, **C** (*chord***)**	**A:** up and down stroke with thumb, twice on each chord, including final chord strummed 4 times **B:** holding the tablet plectrum using the thumb and index, middle or ring finger and strumming across the strings
7	Fine motor control of fingers or arm: single or two finger combinations using various fingers, or pinch grip **Shoulder stabilization, finger extension (any/all finger/s or part of finger), wrist deviation,** elbow extension, wrist extension	**Tablet and Garageband** with “Smartpiano,” sustain switch on, chords set to G (Left side of screen) and G, D5, G in centre of screen, speaker connected. Alternatively, Smartguitar using the plectrum	In line with affected side. At a height approximate to a standard keyboard height, or that does not demand participant to extend arm excessively to reach the keys In some instances placing the tablet on the participant's lap or a table (affected side) may offer the most accessibility and focus on finger extension	Relax shoulders, reach with your finger/s, feet flat as possible on floor Participants may initially trigger the sounds using knuckle or other part of finger The therapist should encourage and support use of finger tips where possible	tablet: playing a single “G” chord by sliding fingertip/s, knuckle, etc., vertically upwards over the chord and/or slightly away from body Alternating affected and unaffected side, 1^st^ beat of each bar	Frere Jacques in G major with strong pulse	**A:** unaffected fingers play the G, D5, G, chords on “ding, dang, dong” lyric section of song **B:** affected side plays G, D5, G section **C:** affected hand uses index, then middle finger to play “G” unaffected hand plays G, D5, G section **D:** using the tablet plectrum and Smartguitar
8	Fine motor control: single to 4 finger combinations **Finger movements or finger extensions**, shoulder stabilizing, elbow flexion and extension, shoulder extension, abduction and adduction. Core muscles	**2 X tablets**: both mounted on a single microphone stand Garageband music software using “Smartpiano.” Tablets connected to speaker using two mini jack leads and a splitter input	Tablets one above the other or next to each other, mounted on two tablet clamps which are both on the same microphone stand	Initially, therapist instructs participants whilst playing the chords by singing the chord names or “play” each time the participant is required to play the next chord.	Any finger or finger combination playing each chord individually in time to the music. Finger can extend to play ascending chord or flex to play descending chord (down the screen)	8 chord sequence played to accompany the alternating chords on tablet 1 and tablet 2 as follows: C, G, Am, Em, F, G, C, C. with each chord arpeggiated. Clear accents on bass note of each chord to emphasize pulse	**A:** single finger extension alternating left and right hands **B:** single finger extensions, affected hand only **C:** 2 fingers alternating left and right hands (index L, index R, middle L, middle R, etc) **D:** alternating left and right hands using 3 fingers (as above) **E:** alternating left and right hands using 4 fingers (as above)
9	Fine motor control: single to 4 finger combinations or thumb and finger pinch grip using tablet plectrum **Finger movements or finger extensions,** Thumb, Index, middle and ring finger extension and flexion, shoulder stabilization, elbow flexion, core muscles	**Tablet:** Garageband music software, “Smartbass” or “Smartguitar” and speaker, mounted on stand or resting on lap or table top, tablet plectrum	Tablet positioned in portrait rather than landscape in order to facilitate “keyboard” style finger patterns on the strings. Position on stand, lap or table top	Relax shoulders, feet flat as possible on floor Initially, sing each chord name	Playing on the first beat, then 1 and 3, then 1, 2, 3.	Chord sequence: C. G. Am, Em, F, G, C:||4/4, (one chord per bar)	**A:** index **B:** index and middle **C:** index, middle ring **D:** index, middle, ring, little finger **E:** thumb, index and other fingers **F:** using tablet plectrum held using thumb and index, middle or ring finger and used to play individual notes on each string or chords
10	Fine motor control: single to 2 finger combinations **Finger movements or finger extensions,** finger, wrist, elbow or shoulder extensions, some shoulder abduction or adduction depending on positioning	**Tablet:** mounted on the boom stand, speaker connected. ThumbJam Cello, E3 to E4, major scale setting	Mounted on stand in portrait position, so that as the fingers move up the screen, the scale ascends	Keep finger in contact with screen for the full duration of the music, moving it up/changing finger in time with the music Sing “play” to participants each time they are required to move to the next note	Playing up the screen: E, F#, G#, A, B, C#, D#, E	Ascending scale: E, B, E, A, E, A, B, E. Descending scale: E, B, A, E, A, E, B, E. Arpeggiating the chords and with a “turn around” phrase at the end to indicate “back to the start note (E)” For example the notes: b, c#, d# on the “B” string	**A:** hold position sustaining the top “E” note of the scale at the top of the screen, then following the music to descend back down the screen to bottom note “E”
11	^1^(Baker et al., 2006) **Grip, wrist ulnar and radial deviation**, elbow flexion	**Cabasa**: small, medium and large sizes **Bongos or cymbal** on stand	Cabasa held in unaffected hand, affected hand aims to grip the beads over the top/round the side with any finger/thumb combination and twist to produce sound	Relax shoulders, “twist, release, reach, play” (spoken or sung to participants in-time with music)	Playing on 1^st^ (cabasa twist) and 3^rd^ (bongo/cymbal) beat of each bar	Strongly pulsed, rhythmic and staccato music, for example Spanish idiom using E, F/E bass, 4/4 time	**A:** grip and twist cabasa with affected hand on beat 1, then release it and hit the cymbal on beat 3 using the affected hand (no stick or beater)
12	**Wrist and forearm pronation and supination,** shoulder adduction, elbow extension, core muscles	**Cymbal on boom and bongos on stand,** two drum sticks or beaters taped together so there is a tip at either end or single stick/beater rotated so that tip plays cymbal then bongo	Cymbal and bongos slightly less than a beater's distance apart focusing efforts on wrist and forearm rotation Alternatively, play bongos, rotating wrist/forearm to play bongo 1 then bongo 2	Sung prompt: turn and turn and play	Affected hand only plays bongo with one end of the stick, then cymbal or bongo 2 with the other end on beat 3 only (slow rotation) or 1 and 3	Octaves or chords: F slide to C, slide to Gm, slide to Dm 4/4 time over 2 bars, with strong accent on beats 1 and 3 and crescendo between each chord	**A:** holding just the adaptive beater and making full forearm pronation and supination movement to play cymbal then bongo or bongo 1, bongo 2 **B:** using a cabasa instead of stick/beater

*Patterns gradually increase in complexity; 1–5, gross motor; 6–10, fine motor; 11 and 12, grip and release, pronation and supination*.

A Go/No Go *task* will be performed to track improvements in reaction time (measuring participant's response to visual stimuli with a button press) and changes in contingent negative variation (CNV) between signals as a marker of attention processes. We expect the reaction time to have shortened more in the early intervention group.

## Interventions

### Therapeutic instrumental music performance (TIMP)

TIMP, a NMT technique which has not been widely researched, is a defined intervention for upper limb rehabilitation, which comprises three essential elements: (1) Musical structure: clearly pulsed music, with melodic, harmonic and dynamic structures, which cue the organization of movements in time, space and force dynamics; (2) Choice of instruments and mode of playing; (3) Positioning or spatial arrangement of instrument/s to facilitate the target movement/s (Thaut, 2008; Thaut and Hoemberg, 2014). TIMP is an intervention that can be delivered following specialist neurologic music therapy training. It involves playing musical instruments or digital music equipment in a way that demands specific movement patterns. Musical equipment is positioned to practice those target movements that patients find difficult, for example elbow flexion and extension (reaching and playing a cymbal), or shoulder abduction (playing a drum to the side of the participant). Specific qualitative aspects of movement such as trajectory smoothing and variability, priming, timing, and movement range are targeted and the music prepared for this study, which can be performed live by the therapist or played in identical, pre-recorded format from a tablet, supports these aspects. All music is set to a metronome beat and each musical pattern that accompanies each exercise is comprised of strongly pulsed, simple repeated patterns, which provide a predictable temporal framework within which participants are able to plan and execute each movement, achieving a high number of repetitions. The aim of the music is to provide an auditory mirroring of movement patterns using melodic contour to support movement direction, and tempo, which is set to the existing speed of participants' movements (see Figures A1–A3 in Supplementary Materials Section).

### Instrument choice

**Figure 3:**
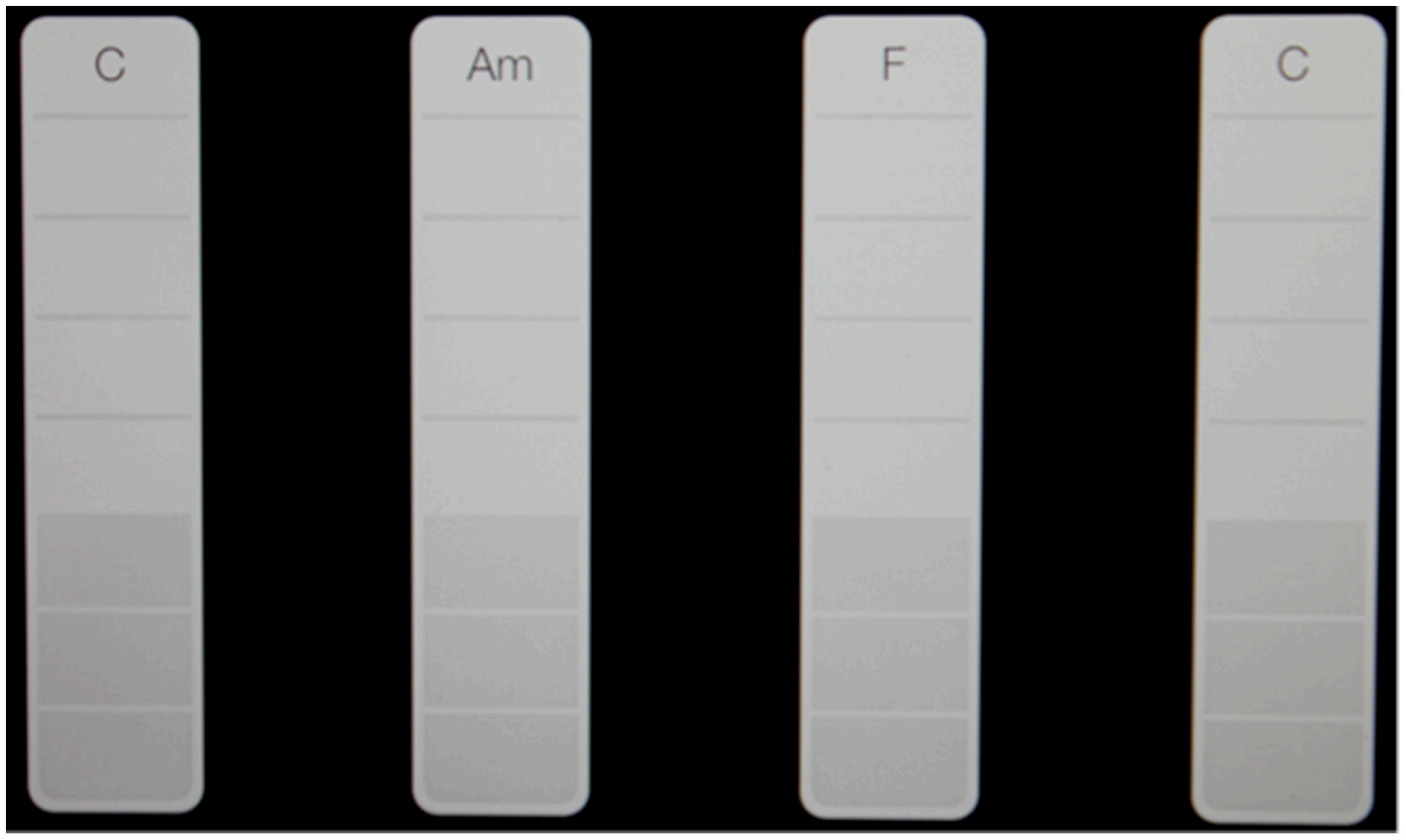
**Chord spacing for the “Smart Piano.”** Participants aim to touch the white strips at the bottom. As they move their finger up, the notes of the chord are sounded.

The instruments played by participants in this study have been selected for their portability, flexibility in offering various spatial arrangements, and the quality and range of audio feedback that they can offer. These are important considerations for a treatment that is being delivered in the home environment, where access and space might prevent the use of many conventional acoustic instruments. Percussion instruments are accessible to non-musicians and require a wide range of movements and movement sequences, potentially employing all muscle groups (Thaut, 2008). They can also be positioned for unilateral and bilateral playing, and played using hands, fingers and other finger joints such as the knuckles, or with beaters and drum-sticks. There is also a playing pattern that facilitates grip and release finger movements (see pattern 11 in the TIMP chart, using hand held percussion). Computer tablet touch screen instruments are also accessible to non-musicians and offer the appeal of more contemporary sound-worlds, with which some participants may identify and be more motivated by than with the acoustic instruments. Audio feedback and quality from the tablet touch screen instruments will be enhanced through the use of a “Jawbone Jambox” Bluetooth, wireless speaker, mounted on the microphone stand that holds the tablets; also saving space and eliminating the need for cables (with the exception of TIMP 8, which requires two tablets). The speaker is extremely resonant and will also be used to provide tactile feedback by placing it on table surfaces as participants play exercises whilst seated at a table. The touchscreen instruments and speaker will not provide the same quality or degree of tactile and acoustic feedback as acoustic instruments, but for this study they were considered to be most suitable to meet the need for a wide enough range of visual targets for fine motor exercises, whilst being portable and offering a variety of motivating instrumental sounds. Playing techniques for tablets do not require technique acquired through musical training and are easily accessible using finger tips, finger and thumb joints and movements not commonly associated with the sounds that they produce; such as that of the “smartpiano,” which requires fingers to be moved vertically up and down across bars on the screen that represent and produce piano chords (see Figure 3), with the bass notes in the lower portion of each bar. Playing these touch screen instruments also requires more shoulder stability and controlled upper limb abduction, adduction, flexion and extension movement patterns than is the case with the larger acoustic instruments which have much larger target areas that are easier to hit.

### Equipment

The instruments used in this study will be: bongos on an adjustable stand, 14″ cymbal on a boom stand, two computer tablets, which mount on a single microphone boom stand using two clamp holders that can present various angles for playing, a small Bluetooth speaker, which mounts on the boom stand with the tablets, Garageband music software, ThumbJam music software, three cabasas (small, medium, and large), a selection of standard and adapted drum sticks and beaters, a pair of drum sticks made for playing tablet touch screen drums, a set of finger picks, a plectrum made for playing tablet touch screen guitar.

Adjustments to the tablet settings (see Appendix in Supplementary Materials) will be made in order to ensure that when participants play the touch screen instruments, the screen does not change or move, but the instrumental sounds are triggered, thus alleviating any frustration that may be caused by technical issues with the tablet on top of participants' existing motor control problems.

Smartpiano chords offer opportunities for participants to practice finger flexion and extension and other fine motor movements using a wide range of finger combinations, including thumb only (see patterns 6–10 in the TIMP chart). Smartbass and Smartguitar will be used to practice these movements, in addition to pinch grip by holding the tablet plectrum and strumming notes and chords. The “sustain” switch for Smartpiano will be set to “on” in order to provide participants with more sustained harmony and auditory feedback before they go on to play the next chord. Chords for the touchscreen instruments (selected from the Garageband instrument menu) will be set for some patterns so that each one is separated by a blank chord space in order to minimize error in participants' playing (see Figure 3).

### Spatial arrangement set-up time and transportation of the instruments

A total minimum area of two meters squared will be required to set up the cymbal on boom stand and bongos on stand, including space for the participant to sit. The area increases depending on how far the instruments will be moved back from or to either side of the participant in order to facilitate greater range of upper limb movements.

Fifteen minutes are required for setting up all equipment, then a further 15 min for packing away, making a total of 30 min setting up time for each session in addition to the time for intervention. It is important to ensure that all equipment is transported with minimum risk of damage when carried in and out of the car and in to the various properties visited. A travel bag with extending handle and wheels will be used to transport the bongos, cymbal and all hardware, beaters and drum sticks. All together this weighs 16.7 kg. A shoulder bag will be used to transport the two tablets, metronome and all paperwork. The microphone stand with tablets brackets attached will be carried separately without cases and classical guitar will be transported in a robust, hard case.

### Using the TIMP chart

The extensive detail for TIMP intervention presented here (Table 1) was developed and refined through the course of delivering treatment to a volunteer stroke patient. It therefore represents an intervention that was refined through patient collaboration, prior to recruiting participants via the host NHS trust database, in order to maximize patient compliance.

Based on the TIMP chart, all musical patterns, which will be played live by the researcher to support participants whilst they play the instruments, have also been recorded onto one of the tablets, using “Garageband” music software. They were recorded at a metronome setting of 50 and 60 beats per minute (bpm) respectively, using the “audio recorder” selected from the Garageband instrument menu and input via the tablet built-in microphone. This offers two tempo settings for participants to try each exercise whilst the therapist physically guides arm movements in cases where hand-over-hand support is required. Following this, the researcher and participant will play together, with the therapist playing the supporting music live, in-time to a metronome, listening via an ear piece, that is adjusted to a tempo which supports each participant's current frequency of movement.

Most of the TIMP patterns have variations, where participants will follow alternative finger patterns, or be given various beaters, drum sticks, plectrums and finger picks to use with the instruments as required. These equipment serve one of two functions: they either facilitate improved access to the instruments and improved sound quality and auditory feedback from their movements and playing, or they require from the participant more complex finger movements, bilateral playing patterns and additional grasp, grip or pinch movements. Some participants may struggle to grip beaters initially and be more able to access instruments using hands and fingers only, with the focus more on gross motor movement. In this way, the musical tasks demand maximum physical performance.

### Metronome settings

Pacing of movements can be problematic in hemiparetic movement disorders. Tapping exercises to external precisely paced auditory cues provide opportunities for rehearsal of movement timings. Using a metronome with a “tap” facility, each participant's playing tempo can be calculated by tapping into the metronome in-time with their playing. Following this, the researcher plays the music in a manner that strongly accents each beat. If the movements involve a high level of compensatory movements, for example from the trunk or shoulder, then the metronome speed will be reduced and the pulsed music played more slowly until the participant can be observed as having more time to plan movements between each beat, and move (playing the instrument/s) in-time with the music, or with more controlled and better quality movements. Once the performance of exercises is seen to become more fluent and the timing of playing more in-keeping with the music, then increases of approximately 10 bpm can be made provided that the movement quality is not compromised. For further reference to tempo and motor learning refer to (Massie and Malcolm, 2012; Furuya et al., 2013).

### Monitoring patient performance

Initially, each exercise will be played by participants for periods of up to 2 min (a timer will be used), after which the researcher will stop and ask the participant if they would like to continue or have a rest. The researcher will also ask more specific questions to determine if the participant is experiencing any discomfort or pain possibly related to each exercise, for example in the back, neck, shoulder, elbow, wrist, fingers, not normally present. If the participant feels that they are experiencing pain or discomfort related to the musical exercises then treatment will be paused and these symptoms discussed, before either continuing or considering any potential need for a GP or physiotherapy consultation.

For all exercises participants will be encouraged to keep their feet flat on the ground in order to provide support for their back and core muscles and optimize movement control when playing the instruments. This instruction to participants has become a part of the TIMP protocol for the study following review of video footage with members of the host NHS trust team and academic supervisors, which was taken during sessions with a volunteer stroke participant prior to this study.

## Discussion

At the center of this study is the aim of testing whether the 12 different TIMP playing patterns (Table 1) and their variations, which have been developed following the TIMP protocol, can be effectively delivered in the home environment and improve upper limb function across a small sample size of participants with hemiparesis following stroke. The TIMP protocol, whilst sharing some attributes with MST and stemming from scientific research into the effects of rhythm on movement kinematics, has not been clinically or scientifically researched to a great extent. Whilst MST uses protocolized musical exercises, it has not explored any additional effects of using rhythm and music, which would be derived from existing scientific evidence for its role in supporting the priming, timing, trajectory and muscle force requirements for the upper limb movements within each exercise pattern.

Research into the effects of musical instrument playing and rhythm supporting movements has been based on a model of daily treatment, 5 days per week, which has produced statistically significant results (Schneider et al., 2007; Altenmüller et al., 2009; Malcolm et al., 2009b; Rojo et al., 2011; Amengual et al., 2013). Studies with a lower frequency of treatment have not been widely conducted and with such a reduction in frequency it is not known what the treatment effect will be.

There is great heterogeneity of upper limb impairment within this patient group and the ARAT has been developed as a tool that can capture change within these parameters by recreating a protocol combining tasks commonly performed within ADLs. The table of TIMP exercise patterns (see Table 1) developed for this study describes the target arm movements for each instrumental exercise in the first column, then the instrument/s and equipment to be used, the positioning of each instrument and how it should be played. It can be seen that the musical exercises require arm, hand and finger movements that are the same or similar to those required in order to perform tasks in the ARAT and 9HPT.

ARAT and 9HPT data will not inform about treatment effects on audio-motor coupling and neural re-organization as demonstrated in other studies with fMRI, TMS, and EEG (Altenmüller et al., 2009; Rojo et al., 2011; Rodriguez-fornells et al., 2012). In order to estimate the feasibility of neurometric EEG measures (John, 1989) as an imaging tool for determining cerebral changes related to the TIMP intervention we plan to visualize audio-motor coupling (Rodriguez-fornells et al., 2012) with the continuous EEG using one participant from each group. A mathematical solution of the inverse problem of EEG sources allows the creation of a low resolution electromagnetic tomography (LORETA) of brain regions (Pascual-Marqui et al., 1994) and we can correlate the estimated EEG source activity of the raw and *z*-scored transformed means (Thatcher et al., 2005). EEG cannot visualize brain activity of the midbrain but of the cortex and this is where we expect most lesions after stroke.

Utilizing these imaging tools we also plan to explore a comparison of differences between early and late intervention. Two clients will be subjected to an EEG but we are aware that the imaging results will not be as high resolution as those provided by fMRI. Furthermore, with only two participants, no resulting statistical differences are sought or expected. EEG measures changes in electrical current in the brain and has been utilized in studies on the recovery of stroke patients (Giaquinto et al., 1994), prefrontal-to-motor cortex connectivity (Picazio et al., 2014), post-movement beta-event-related-synchronization (PMBS) in stroke patients with mild hemiparesis (Eder et al., 2006) and current stroke studies utilizing MST in stroke rehabilitation (Altenmüller et al., 2009; Rodriguez-fornells et al., 2012). To explore the limitations and advances of the imaging techniques proposed for this study, the manageability of the measurement process and the cost-effectiveness of utilizing low-cost, portable EEG apparatus and analysis, compared to lab-based, more expensive fMRI measures is a legitimate goal for a feasibility study.

Thus, the intention of this feasibility study is to provide and test a platform, via the TIMP playing patterns, for breaking down movement sequences, facilitating a high level of repetition of specific movements within an activity that is interactive and enjoyable and that is clearly linked to movements required for ADLs. Exercises are performed within clearly structured and repeated rhythmic, musical frameworks, the like of which are evidenced as potential drivers of neural reorganization specifically in the realm of stroke hemiparesis rehabilitation, and also found to reduce learned misuse or compensatory motor behaviors.

Although in this feasibility study, with a small sample size, we do not predict significant group outcomes, we still expect to report on feasibility of the delivery and efficacy of the intervention. We are not intending to apply non-parametric statistical analysis and aren't expecting larger generalizability of the data (which would be increased by applying non-parametrical testing) but want to explore tendencies achievable with parametric data analysis strategies, research design and conditions that would apply with a larger and powered sample size.

MST and trials investigating the effects of rhythm and music on upper limb kinematics have taken place in research laboratories and included, predominantly, inpatients 2 months post stroke. This TIMP study includes participants up to 5 years post stroke, where community rehabilitation in their home has been completed. The majority of rehabilitation for stroke patients in the UK takes place in patients' homes and does not target upper limb hemiparesis alone, but mobility and independent living skills in a more holistic model. To date there have been no reports on feasibility for this type of intervention in participants' homes. As such the study will make a contribution to new knowledge in the field that could influence future service design.

## Author contributions

AS developed the treatment protocol following the TIMP guidelines set out by Michael Thaut, conducted the literature review and drafted the manuscript. HO, WM, and JF advised on the initial overall design, ethics, and timing of clinical and research protocols. JF advised on feasibility of treatment frequency and sample size. JF, WM, and HO edited draft manuscripts and advised on structure and content. AB facilitated the hosting of the study, advised on recruitment sites and procedures and enabled blind assessment.

### Conflict of interest statement

The authors declare that the research was conducted in the absence of any commercial or financial relationships that could be construed as a potential conflict of interest.

## Footnotes

^1^This exercise is described and illustrated in Baker et al. (2006), p79 and adapted for this exercise.

## Abbreviations

TIMP: Therapeutic Instrumental Music Performance
MST: Music Supported Therapy
RAS: Rhythmic Auditory Stimulation
NMT: Neurologic Music Therapy
ARAT: Action Research Arm Test
9HPT: Nine Hole Peg Test
Bpm: Beats per Minute
CCS NHS Trust: Cambridgeshire Community Services National Health Trust
ADLs: Activities of Daily Living.
