# Supplementary material for: Home-based neurologic music therapy for upper limb rehabilitation with stroke patients at community rehabilitation stage—a feasibility study protocol
Source: Front Hum Neurosci. 2015 Sep 23;9:480. doi: 10.3389/fnhum.2015.00480 (PMC4585041; doi:10.3389/fnhum.2015.00480)
Supplement: Supplementary file 1 [file DataSheet1.DOCX]

**APPENDIX**

**
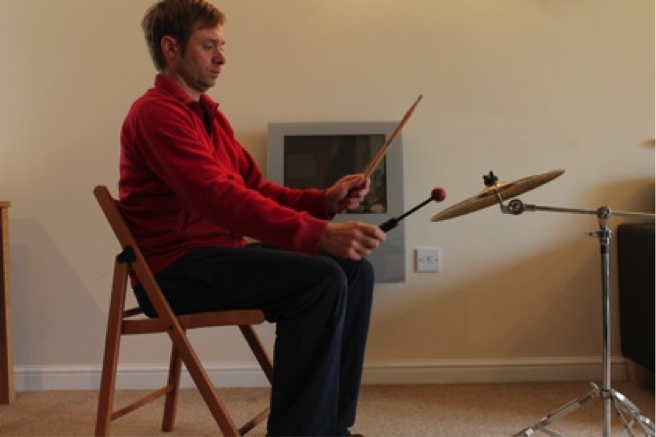
**

**Figure A1. The researcher playing TIMP pattern 1c, bilateral playing for elbow extension.**

**
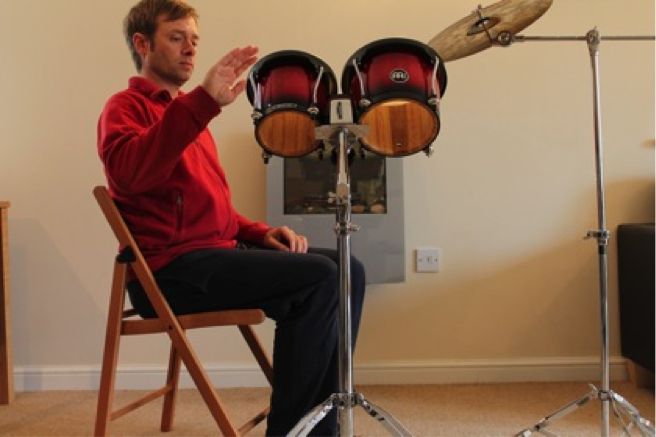
**

**Figure A2. The researcher playing TIMP pattern 2, shoulder abduction.**

**
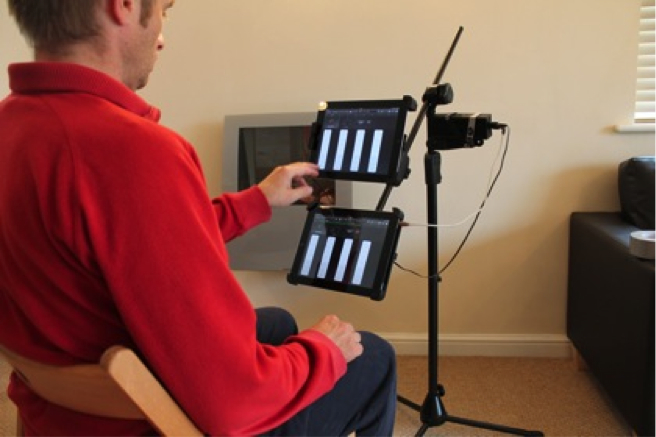
**

**Figure A3. The researcher playing TIMP pattern 8, fine motor control. Single to all finger combinations using two tablets and speaker on stand.**

**Guide for using tablets**

Before setting up tablets for participants to play, settings need to be adjusted under ‘SETTINGS’ from the main screen menu as follows:

1. 'auto-lock’ set to 'never'
2. 'lock rotation' whilst holding the tablet in landscape position
3. 'multitasking gestures' off

Also within SETTINGS, in the ‘control center’, ‘Access Within Apps’ needs to be disabled. This will then prevent the ‘control center’ menu from popping up when participants play the smart keyboard beginning with upwards finger movements on the chord bars from the bottom of the screen.
